# From Integrated Analysis to Clinical Insight: ncRNA-Mediated Ferroptosis in Glioblastoma

**DOI:** 10.3390/cancers18081238

**Published:** 2026-04-14

**Authors:** Venkata N. Seerapu, Rajalakshmi Amaresan, Udhayakumar Gopal

**Affiliations:** 1 Department of Surgery, University of Mississippi Medical Center, Jackson, MS 39216, USA; vseerapu@umc.edu; 2 Department of Zoology, Auxilium College, Gandhi Nagar, Vellore 632 006, Tamil Nadu, India; rlakshmi@auxiliumcollege.edu.in; 3 Department of Neurosurgery, Huntsman Cancer Institute, Salt Lake City, UT 84112, USA

**Keywords:** glioblastoma, ferroptosis, noncoding RNA, iron metabolism, lipid peroxidation, multi-omics

## Abstract

Glioblastoma (GBM) is the most lethal and aggressive primary brain tumor in adults. Despite a standard-of-care regimen involving surgical resection, radiotherapy and temozolomide (TMZ), median overall survival typically hovers between 12 and 15 months. This poor prognosis is driven by profound intratumoral heterogeneity, glioma stem cell populations, and an immunosuppressive microenvironment that collectively fuel resistance to traditional apoptosis-centric therapies. Ferroptosis—a form of regulated cell death driven by iron-dependent phospholipid peroxidation and the collapse of antioxidant defenses—has emerged as a compelling alternative for eliminating therapy-refractory GBM cells. This review examines the molecular machinery of ferroptosis in glioma and explores how an additional regulatory layer, noncoding RNAs (ncRNAs), modulates this process. We highlight key experimentally validated axes where microRNAs, long noncoding RNAs (lncRNAs), and circular RNAs (circRNAs) orchestrate iron handling and antioxidant thresholds. These include sensitizers like miR-147a and circLRFN5, which promote iron overload, and resistors like circCDK14 and TMEM161B-AS1, which act as “ferroptosis brakes”. Furthermore, we discuss how integrative analyses of TCGA and CGGA cohorts have yielded ferroptosis-related lncRNA signatures that robustly predict patient survival. Finally, we outline the clinical potential of these ncRNAs as biomarkers and therapeutic targets while addressing the delivery challenges, such as the blood–brain barrier, that must be overcome to achieve precision, ferroptosis-oriented GBM therapy.

## Simple Summary

Glioblastoma is an aggressive brain cancer that is very hard to treat, and most patients live little more than a year despite surgery, radiation, and chemotherapy. Scientists are now studying a special type of cell death called ferroptosis, which kills cancer cells by overloading them with iron and damages their fatty cell membranes. Tiny genetic switches called noncoding RNAs (which do not make proteins) help control how easily glioblastoma cells undergo ferroptosis by regulating iron levels, antioxidant defenses, and stress responses. Some of these RNAs protect tumor cells from dying, while others make them more vulnerable. By combining large-scale data analysis with laboratory experiments, researchers are mapping these RNA networks and linking them to patient survival and treatment response. In the future, this knowledge could be used to predict which patients will respond to therapy and to design new treatments that push glioblastoma cells into ferroptosis while sparing healthy brain tissue.

## 1. Introduction

### 1.1. Glioblastoma and Need for Novel Modalities

Glioblastoma (GBM) remains the most frequent malignant primary brain tumor in adults, where current treatments offer only modest survival benefits. For those newly diagnosed, first-line treatments involve the “Stupp regimen”—a rigorous combination of maximal safe surgical resection, radiotherapy (typically 60 Gy in 30 fractions) and concomitant daily temozolomide (TMZ). Despite this multimodal approach, outcomes remain grim; median overall survival hovers between 12 and 15 months, and long-term survival is tragically rare. Meanwhile, real-world data confirms that the Stupp protocol is superior to less intensive care, yet most patients still face early progression [1,2,3,4,5,6,7,8,9].

The primary obstacle to lasting control is therapy resistance, a complex problem fueled by genetic, epigenetic, and microenvironmental factors. For instance, TMZ resistance is heavily influenced by O-6-methylguanine-DNA methyltransferase (MGMT) status and DNA repair pathway activation. Furthermore, oncogenic signaling (such as PI3K/Akt/mTOR), the presence of glioma stem-like cells, profound intratumoral heterogeneity, and an immunosuppressive tumor microenvironment work together to thwart radiotherapy, chemotherapy, and emerging immunotherapies. Because recurrent tumors often evolve into molecularly distinct mesenchymal-like entities, the challenge of treating GBM is constantly shifting [5,8,10,11].

### 1.2. Ferroptosis: A New Therapeutic Front

In response to these challenges, ferroptosis has emerged as a compelling regulated cell death modality driven by iron-dependent phospholipid peroxidation and the failure of the glutathione peroxidase 4 (GPX4)-centered antioxidant system. This process is driven by excess ferrous iron generated through transferrin receptor-mediated import and ferritinophagy (e.g., NCOA4-dependent FTH1 degradation) which catalyzes Fenton chemistry. Simultaneously, upregulation of ACSL4 and LPCAT3 enriches the membrane with polyunsaturated phospholipids, providing the essential substrates for lipid peroxidation by lipoxygenases and related oxidases. The system Xc^−^/GSH/GPX4 axis, together with parallel antioxidant circuits such as FSP1–CoQ10, normally detoxifies phospholipid hydroperoxides and restrains ferroptosis, positioning SLC7A11, GPX4, and ACSL4 as pivotal molecular markers. ferroptosis is best conceptualized as a threshold phenomenon in which iron-driven lipid ROS surpasses the buffering capacity of endogenous defense pathways. In GBM this “ferroptosis threshold” is locally tuned by the heterogeneous microenvironment, including hypoxic niches, nutrient/redox gradients, and variable immune and myeloid cell infiltration. Lipid-rich membranes are particularly vulnerable to this collapse because ACSL4 and related enzymes selectively incorporate polyunsaturated fatty acids (PUFAs) into phospholipids, rendering them preferred substrates for iron-catalyzed peroxidation. Distinguished from apoptosis (caspase-dependent, DNA fragmentation), necroptosis (RIPK1/RIPK3/MLKL-driven membrane rupture), and pyroptosis (gasdermin-mediated inflammatory pore formation), ferroptosis is uniquely defined by shrunken, high-density mitochondria and the failure of highly druggable antioxidant bottlenecks [12,13,14,15,16,17,18,19,20,21,22,23,24,25]. This makes ferroptosis a distinct therapeutic vulnerability in GBM, particularly in cells that maintain robust DNA repair and anti-apoptotic programs but remain reliant on high PUFA flux. Within this framework, pro-ferroptotic stimuli act as sensitizers that lower the threshold by promoting iron accumulation, whereas anti-ferroptotic mechanisms function as ferroptosis brakes that raise the threshold by enhancing iron export, boosting GPX4- and BH4-dependent detoxification, or shifting membranes toward less oxidizable lipids.

### 1.3. Noncoding RNAs and GBM Biology

Noncoding RNAs add an additional regulatory layer to these ferroptosis pathways. MicroRNAs (miRNAs), long noncoding RNAs (lncRNAs), and circular RNAs (circRNAs) collectively orchestrate gene expression, influencing nearly every hallmark of GBM. Dysregulated miRNAs modulate oncogenic networks by binding to mRNAs, while lncRNAs act as scaffolds or decoys to sustain GBM stem cell self-renewal. CircRNAs, characterized by high stability, further finetune these circuits by sponging miRNAs. This pervasive involvement of ncRNAs in glioma provides a strong rationale to explore how specific ncRNA networks intersect with iron metabolism and lipid peroxidation [26,27,28,29,30,31,32,33].

### 1.4. Ferroptosis and ncRNA: A Convergence Point

A growing body of evidence now directly links this ncRNA layer to the orchestration of ferroptosis. Ferroptosis-sensitizing ncRNAs like miR 147a and circLRFN5 actively facilitate iron overload and lipid peroxidation: miR 147a achieves this by directly targeting the iron exporter SLC40A1 while circLRFN5 promotes the degradation of the transcription factor PRRX2 and downregulates the GCH1 BH4 antioxidant axis. These actions collectively induce ferroptotic cell death and suppress glioma stemness and tumorigenicity [34,35]. In contrast, oncogenic regulators like circCDK14 and the lncRNA TMEM161B AS1 function as ferroptosis brakes. circCDK14 sustains PDGFRA signaling and preserves SLC7A11/GPX4 expression to confer ferroptosis resistance; similarly, TMEM161B AS1 sponges have miR 27a 3p to upregulate FANCD2 and CD44, thereby supporting DNA repair, stemness, and temozolomide resistance facilitating ferroptotic escape in GBM models [36,37,38]. Beyond individual molecules, integrative analyses of TCGA and CGGA cohorts have yielded multigene, ferroptosis-related lncRNA signatures capable of predicting glioma patients’ prognosis and immune state, underscoring ncRNA-mediated ferroptosis as both a mechanistic driver and a clinically exploitable vulnerability [23,37,39,40].

This review aims to synthesize and critically appraise the extant understanding of ncRNA-mediated ferroptosis in glioblastoma, focusing on how integrated multi-omics analyses can bridge the gap between mechanistic insights into clinically meaningful biomarkers. We begin by summarizing the core molecular machinery of ferroptosis in GBM and outline the major classes of noncoding RNAs—including miRNAs, lncRNAs, and circRNAs—involved in glioma progression. We then highlight pivotal ferroptosis-regulating axes, featuring experimentally supported examples such as miR-147a, circLRFN5, circCDK14, and TMEM161B-AS1, alongside emerging ferroptosis-related lncRNA signatures derived from TCGA and CGGA cohorts. Finally, we examine how these ferroptosis-associated ncRNA networks intersect with the immune microenvironment and therapy response, identify critical gaps in our current translational research, and propose future directions for leveraging ncRNA-directed ferroptosis modulation as a cornerstone of precision medicine in GBM.

## 2. Ferroptosis: Mechanistic Basis in Glioblastoma

### 2.1. Core Hallmarks

**Figure 1:**
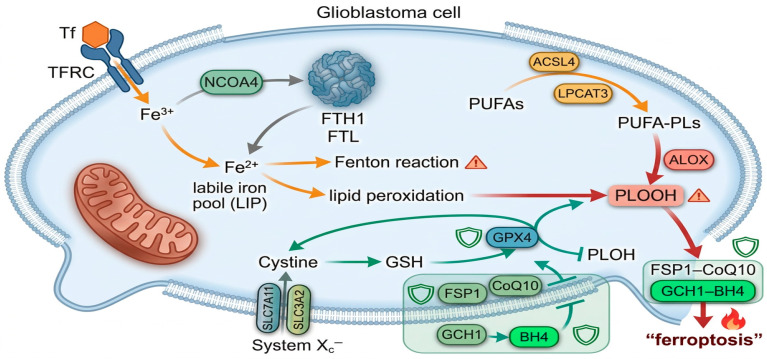
Core ferroptosis pathways in glioblastoma. Iron uptake via TFRC- and NCOA4-mediated ferritinophagy expands the labile iron pool, driving Fenton chemistry and lipid peroxidation of PUFA-containing phospholipids (PUFA PLs) generated by ACSL4/LPCAT3 and oxidized by ALOX. Three antioxidant axes counteract this: GPX4 uses GSH supplied by system Xc^−^ (SLC7A11/SLC3A2) to reduce phospholipid hydroperoxides (PLOOH), while FSP1–CoQ10 and GCH1–BH4 provide parallel radical trapping protection at the plasma membrane. Warm arrows indicate pro-ferroptotic flows, green arrows indicate ferroptosis-suppressive pathways, shield icons mark ferroptosis brakes, and hazard icons indicate sites of toxic lipid peroxide accumulation.

Ferroptosis in glioblastoma is driven by a sophisticated interplay between iron metabolism, lipid peroxidation, and antioxidant defense pathways that together determine cellular sensitivity to iron-dependent cell death [12,13]. GBM cells frequently exhibit “iron addicted” phenotypes, characterized by the upregulated transferrin receptor-mediated iron import and accelerated ferritin turnover. Furthermore, NCOA4-dependent ferritinophagy expands the labile Fe^2+^ pool and fuels Fenton chemistry and subsequent reactive oxygen species (ROS) production. Simultaneously, enhanced expression of ACSL4 and LPCAT3 enriches cellular membranes with polyunsaturated phospholipids that are preferred substrates for iron-dependent oxidases, thereby predisposing GBM cells to catastrophic phospholipid peroxidation. Counterbalancing these pro-oxidant forces, the system Xc^−^/GSH/GPX4 axis and auxiliary pathways such as FSP1–CoQ10 act as central ferroptosis suppressors by maintaining glutathione pools, and detoxifying phospholipid hydroperoxides. Consequently, SLC7A11, GPX4, and ACSL4 have emerged as definitive molecular markers and therapeutic targets in ferroptosis-oriented GBM studies [12,14,15,16,41]. An overview of these interconnected iron, lipid peroxidation, and antioxidant pathways is depicted in Figure 1. Notably, while direct ncRNA-mediated control of parallel axes like FSP1–CoQ10 and GCH1–BH4 remain sparsely documented in GBM, these pathways are recognized as functionally vital “ferroptosis brakes” and represent likely, yet-to-be defined targets for ncRNA regulation.

### 2.2. Validating Ferroptosis in Glioblastoma: A Methodological Framework

Defining ferroptosis in GBM requires a dual focus on its unique biochemical hallmarks, principally iron metabolism and lipid peroxidation—and the rigorous methodological standards required for validation. In GBM models, these experimental readouts converge on three features: iron accumulation, lipid peroxidation, and GPX4-centered antioxidant impairment.

To ensure reproducibility, the current literature recommends confirming ferroptosis through a four-pillar framework:

1. Detection of cell death using LDH release assays or morphological analysis. While GBM cells typically exhibit shrunken, electron-dense mitochondria on electron microscopy, they may also manifest “balloon-bursting” appearances in vitro. Interestingly, though mitochondrial changes are characteristic, most GBM ncRNA studies currently infer ferroptosis from ROS readouts rather than direct ultrastructural evidence—marking a clear avenue for future investigation.
2. Pharmacologic rescue: The specific nature of cell death must be validated by restoring viability with canonical inhibitors like Ferrostatin-1 or Liproxstatin-1, while confirming that caspase blockade (apoptosis inhibition) fails to provide rescue.
3. Lipid peroxidation identification utilizing products like malondialdehyde (MDA) or fluorescent probes (e.g., C11-BODIPY) and mass spectrometry-based epilipidomics.
4. Iron involvement: Verifying the role of the labile iron pool through assays such as calcein-quenching or FerroOrange.

High-quality reviews further emphasize technical rigor: radical-trapping antioxidants (RTAs) should be applied at precise, sub-micromolar concentrations to avoid off-target effects. Furthermore, the environmental context is critical; the metabolic instability of Ferrostatin-1 necessitates the use of Liproxstatin-1 for in vivo studies. Finally, researchers must account for variables—such as cell density and media composition (e.g., selenium levels)—that significantly modulate ferroptotic sensitivity within the GBM microenvironment [20,42,43,44,45,46,47,48,49,50,51,52].

### 2.3. Evidence of Ferroptosis in Glioma

Preclinical studies show that GBM cell lines and patient derived models undergo bona fide ferroptosis in response to system Xc^−^ and GPX4 inhibitors such as erastin and RSL3, with cell death reversed by ferroptosis-specific blockers like Ferrostatin-1 and Liproxstatin-1. Targeting key regulators—including ACSL4, ferritinophagy components (COPZ1/NCOA4/FTH1), and iron handling proteins (TFR2)—modulates lipid peroxidation and enhances temozolomide (TMZ) sensitivity. Combining ferroptosis inducers with radiotherapy or BH3 mimetics potentiates glioma cell death and can overcome classical resistance mechanisms [12,47,50,53,54,55,56,57]. Collectively, these findings highlight ferroptosis as a non-redundant death program; even modest ncRNA-mediated modulation of antioxidant nodes can decisively shift whether a glioblastoma cell succumbs to or escapes therapeutic stress.

## 3. Overview of Noncoding RNAs in Glioblastoma

Noncoding RNAs comprise several mechanistically distinct classes that collectively rewire transcriptional and post-transcriptional programs in glioma. MicroRNAs (miRNAs) are ~22 nt in length and guide the RNA-induced silencing complex (RISC) to target mRNA 3′ UTRs, resulting in the degradation or translational repression of genes controlling cell cycle progression, invasion, and apoptosis. Long noncoding RNAs (lncRNAs), (>200 nt) function as nuclear scaffolds for chromatin modifiers or cytoplasmic “decoys” that sustain glioma stem cell self-renewal and confer chemoresistance. Circular RNAs (circRNAs), formed via back splicing into covalently closed loops, exhibit superior stability and often act as miRNA sponges or protein scaffolds. These pervasive regulatory roles provide a robust mechanistic foundation for investigating how ncRNA circuits intersect with iron metabolism and the GPX4 system to modulate ferroptosis sensitivity [26,27,28,29,30,31,32,33].

### 3.1. miRNAs Regulating Ferroptosis Pathways in Glioblastoma

Several microRNAs have emerged as pivotal regulators of ferroptosis sensitivity in GBM, primarily by modulating iron handling or compromising antioxidant defenses [56,58,59]. miR 147a represents the best characterized sensitizer in this context. Often downregulated in GBM, its restoration suppresses cell viability while increasing intracellular iron accumulation and lipid peroxidation. Mechanistically, miR 147a directly binds the 3′ UTR of the iron exporter SLC40A1 (ferroportin); by repressing ferroportin expression, it effectively blocks iron efflux leading to iron overload-driven death. Importantly, miR 147a mimics enhance temozolomide cytotoxicity in vitro, and SLC40A1 overexpression rescues both ferroptosis and TMZ sensitivity, highlighting a miR 147a/SLC40A1 axis that links iron metabolism, ferroptosis, and chemoresistance in GBM [35,60,61].

**Figure 2:**
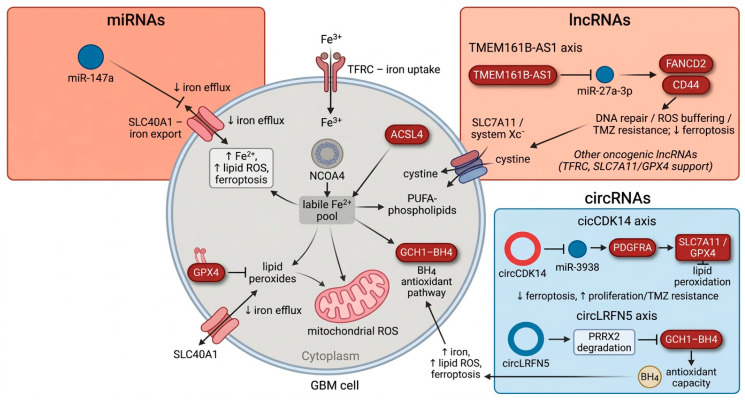
ncRNA-mediated regulation of iron and ROS metabolism in glioblastoma. The central GBM cell depicts key ferroptosis and redox nodes, including TFRC-mediated iron uptake, NCOA4-dependent ferritinophagy, SLC40A1-regulated iron export, ACSL4-driven phospholipid synthesis, and the system Xc^−^/GPX4 and GCH1–BH4 antioxidant axes. Surrounding panels detail specific ncRNA circuits modulating these pathways: (i) miRNAs: miR-147a targets SLC40A1 to impair iron efflux, thereby increasing labile iron and lipid ROS to promote ferroptosis. (ii) lncRNAs: The TMEM161B-AS1/miR-27a-3p/FANCD2-CD44 axis, alongside other oncogenic lncRNAs, stabilizes SLC7A11/GPX4 activity to reinforce ROS buffering and ferroptosis resistance. (iii) circRNAs: circCDK14 sustains PDGFRA signaling and downstream antioxidant defenses to suppress ferroptosis, whereas tumor-suppressive circLRFN5 triggers PRRX2 degradation, attenuating the GCH1–BH4 axis and inducing cell death. Arrows indicate activation; blunt lines indicate inhibition. Red denotes oncogenic/ferroptosis-suppressive ncRNAs, while blue denotes tumor-suppressive/ferroptosis-promoting ncRNAs.

Beyond directly validated GBM regulators, miRNAs characterized in other tumor types provide highly relevant mechanistic templates. For instance, miR 27a 3p is sequestered by the lncRNA TMEM161B AS1 in glioma-targets FANCD2 and CD44, two genes that protect cells against lipid ROS and radiotherapy-induced stress. In line with these functional data, FANCD2 is upregulated in glioma and behaves as a ferroptosis-suppressive DNA repair factor whose inhibition increases ROS, enhances TMZ-induced ferroptosis, and improves prognosis, indicating that the TMEM161B AS1/miR 27a 3p axis reinforces both Fanconi anemia pathway repair and ferroptotic escape via FANCD2 in GBM. Beyond glioma, miR 214 3p has been shown to aggravate ferroptosis by directly targeting GPX4 and modulating the SLC7A11/GPX4/ACSL4 axis in kidney injury models, whereas miR 324 3p reduces GPX4 expression to promote ferroptosis in prostate carcinoma cells, suggesting that analogous GPX4 targeting miRNA circuits could operate in GBM. Notably, bioinformatic and experimental studies report that miR 214 3p and members of the miR 324 family are dysregulated in glioma—miR 214 3p being sequestered by lncRNAs such as HOTAIR in TMZ-resistant GBM cells, and miR 324 5p/3p being generally downregulated in high-grade glioma—supporting their relevance as hypothesis-generating candidates rather than validated ferroptosis regulators in GBM. More broadly, systematic and mechanistic studies across cancers indicate that ferroptosis-related miRNAs frequently converge on central regulators such as SLC7A11, GPX4, ACSL4, and iron transport proteins, implying that miRNA-based manipulation of these nodes may provide a flexible strategy to either enhance or restrain ferroptosis in glioblastoma depending on the therapeutic context [37,56,58,62,63,64,65,66,67,68]. Figure 2 summarizes major ncRNA classes and representative oncogenic and tumor-suppressive transcripts in GBM, emphasizing how they intersect iron handling and ROS metabolism.

### 3.2. lncRNAs Regulating Ferroptosis in Glioma

Long noncoding RNAs regulate ferroptosis both through individual, validated axes and as components of multi-lncRNA risk signatures [37,69,70,71]. TMEM161B AS1 serves as a prototypical oncogenic lncRNA in GBM; its upregulation in TMZ-resistant cells promotes “ferroptotic escape”. By acting as a competing endogenous RNA (ceRNA) for miR-27a-3p, it de-represses FANCD2 and CD44, thereby stabilizing a FANCD2-dependent brake on ferroptosis and strengthening DNA repair pathways. Consistent with this view, independent analyses show that FANCD2 is overexpressed in glioma, correlates with a higher grade and worse outcome, and functions as a ferroptosis suppressor—underscoring that TMEM161B AS1 promotes TMZ resistance not only by strengthening FA pathway DNA repair but also by stabilizing a FANCD2-dependent brake on ferroptosis [37,65,66,72,73].

Complementing these single-gene studies, integrative analyses of TCGA and CGGA cohorts have utilized Weighted Gene Co-expression Network Analysis (WGCNA) to construct ferroptosis-related lncRNA (FR-lncRNA) signatures. Another FR lncRNA risk score built from eight or nine prognostic lncRNAs in lower grade glioma (LGG) showed strong, independent prognostic power across TCGA, CGGA, and Gravendeel datasets and was associated with MGMT methylation, IDH status, and radiotherapy benefit, implying that ferroptosis-related lncRNA programs influence both intrinsic tumor biology and treatment responses. These multivariate panels robustly stratify patients by overall survival and molecular subtype. High-risk scores in these models frequently correlate with immunosuppressive microenvironmental patterns, suggesting that lncRNA-mediated ferroptosis programs influence not only intrinsic tumor biology but also the broader immune landscape [69,70,71,74].

### 3.3. circRNAs Regulating Ferroptosis in Glioblastoma

circRNAs have emerged as critical modulators, with circCDK14 and circLRFN5 representing two contrasting archetypes. circCDK14 is significantly overexpressed in glioma and correlates with poor prognosis. It functional studies show that circCDK14 promotes glioma cell proliferation, migration, invasion, and tumor growth, while its knockdown suppresses malignancy and enhances sensitivity to erastin-induced ferroptosis. Mechanistically, circCDK14 acts as a ceRNA for miR 3938 to de-repress PDGFRA, which sustains downstream SLC7A11 and GPX4 expression, effectively limiting lipid peroxidation [12,36,40,75].

In contrast, circLRFN5 acts as a tumor suppressor that enforces ferroptotic vulnerability. circLRFN5 is downregulated in GBM specimens and glioma stem-like cells, and restoring its expression inhibits proliferation, invasion, and self-renewal while reducing tumorigenicity in vivo. It facilitates the degradation of PRRX2, which in turn suppresses the GCH1–BH4 antioxidant pathway. This leads to classic ferroptotic phenotypes that can be rescued by ferroptosis inhibitors but notably not by inhibitors of apoptosis or necroptosis. Together, circCDK14 and circLRFN5 illustrate how circRNAs can either buffer against or drive ferroptotic stress in GBM by rewiring growth factor signaling and antioxidant defenses, positioning circRNA-centered axes as attractive targets for ferroptosis-based therapeutic strategies [23,34]. In Table 1, we summarize representative ferroptosis-modulating ncRNAs—miR 147a, TMEM161B AS1, circCDK14, and circLRFN5—highlighting their expression patterns, targets, ferroptosis effects, and therapeutic relevance. Taken together, these examples indicate that ncRNA–ferroptosis interactions in GBM can be distilled into a limited set of recurrent regulatory patterns: (i) ncRNAs that tune iron handling and the labile Fe^2+^ pool (for example miR 147a- and FANCD2-linked lncRNAs), (ii) ncRNAs that shape PUFA phospholipid metabolism and lipid peroxidation through nodes such as SLC7A11/GPX4 and ACSL4, and (iii) ncRNAs that reinforce or dismantle antioxidant ‘brakes’ like the GCH1–BH4 and GPX4 systems. These pathway-level motifs provide a mechanistic scaffold for interpreting multi-omics ferroptosis-related lncRNA signatures as systems-level extensions of the same regulatory architecture rather than as independent prognostic constructs.

## 4. Integrated Analyses of Ferroptosis-Related ncRNAs in Gliomas

Building on these recurring regulatory patterns, researchers have transitioned beyond single-molecule studies toward large-scale transcriptomic and clinical analysis to map these networks at a system level. Most studies leverage bulk RNA-sequencing (RNA-seq) data from the TCGA and CGGA databases, correlating lncRNA expression with curated ferroptosis-related gene sets. By applying least absolute shrinkage and selection operator (LASSO) and Cox regression, researchers have derived multi-ncRNA risk scores that stratify patients by clinical outcome and microenvironment landscape [69,70,71,76]. This section emphasizes the clinical utility of these integrated models, particularly regarding risk stratification and immune contextualization.

One representative study analyzed over 14,000 lncRNAs in the TCGA and 1000 in CGGA, identifying several hundred ferroptosis-related lncRNAs candidates. From these, a 15 lncRNA ferroptosis-related lncRNA signature (FRLS) was constructed. This FRLS robustly predicted overall survival across TCGA, CGGA, and REMBRANDT cohorts and remained an independent prognostic factor after adjustment for age, grade, and IDH status. High risk scores were significantly associated with more aggressive features including WHO grade IV, IDH wild type status and MGMT unmethylated promoters, suggesting that ferroptosis-linked lncRNA programs are intertwined with canonical prognostic markers. Notably, high-risk groups frequently exhibit increased expression of immune checkpoints such as PD 1, PD L1, CTLA4—indicating that a pro-ferroptotic lncRNA signature often coincides with a highly immunosuppressed microenvironment [39,71,74].

Complementary work using Weighted Gene Co-expression Network Analysis (WGCNA) identified 30 hub lncRNAs that overlapped across modules for ferroptosis, tumor progression, and microenvironment. This panel, validated via qRT PCR in independent cohorts, showed strong associations between high-risk scores and macrophage-associated chemokines. Additional signatures in lower grade glioma (LGG) have identified 7 to 11 lncRNA panels that similarly correlate with iron transport and immune-related gene sets, reinforcing the view that these networks capture both intrinsic tumor biology and microenvironmental remodeling [69,70,71,74,76,77].

Collectively, these integrated analyses reveal that ferroptosis-associated ncRNAs are not isolated regulators but components of broader risk modules [69,70,71,77]. However, a critical limitation remains: these signatures are primarily correlative. Most component lncRNAs were selected based on co-expression patterns, yet only a minority have been functionally validated for direct effects on iron handling or the GPX4 system. Thus, current FR-lncRNA panels are best viewed as hypothesis-generating, systems-level markers of ferroptosis-linked transcriptional states. Future research must integrate multi-omics with functional screening to transition these molecules from correlative biomarkers to causal therapeutic targets in glioblastoma.

## 5. Clinical Implications and Therapeutic Opportunities

Building on the mechanistic axes and integrated signatures outlined above, we now consider how these insights can inform target prioritization and exploratory frameworks for patient stratification. The intersection of ferroptosis and ncRNAs offers a “dual lever” for personalized oncology, providing novel avenues for both molecular diagnosis and targeted intervention [53,56,70,71].

### 5.1. Biomarkers and Patient Stratification

Ferroptosis-related lncRNA signatures (FRLS) currently function as potent prognostic tools that complement standard clinical factors. Rather than merely listing validated drivers these signatures reflect complex ferroptosis-associated tumor states [70,71,78]. Multi-lncRNA ferroptosis signatures stratify patients into high- and low-risk groups with significantly different overall survival, independent of age or IDH status [71,78].

Notably, high-risk FRLS scores are frequently associated with IDH wild type status, MGMT-unmethylated promoters, and more aggressive molecular subtypes [70,71]. Because these signatures also correlate with immune cell composition, and checkpoint expression (e.g., PD 1, PD L1 and CTLA4), they may serve as a critical triage tool. A high FRLS score could flag patients for clinical trials combining ferroptosis inducers with immune checkpoint blockage, whereas low-risk patients may be best served by the standard Stupp protocol without the added toxicity of experimental treatments [39,53,70,71,74]. Furthermore, while liquid-biopsy assays for circulating exosomal ferroptosis-related ncRNAs are not yet systematically validated in GBM, they represent a promising non-invasive frontier for monitoring treatment response [26,79,80].

### 5.2. Therapeutic Modulation of Ferroptosis via ncRNAs

The ncRNAs discussed—specifically miR 147a, TMEM161B AS1, circCDK14, and circLRFN5—are inherently druggable using current RNA-based technologies. Restoring sensitizing miRNAs (e.g., miR 147a) or inhibiting suppressive miRNAs could enhance iron overload and lipid peroxidation, synergizing with TMZ [35,53]. Similarly, targeting oncogenic lncRNAs like TMEM161B-AS1 with antisense oligonucleotides (ASOs) or siRNAs could downregulate the FANCD2/CD44 axis, effectively lowering the ferroptosis threshold [36,37,73]. This ncRNA-guided approach allows for pathway-specific selection: clinicians might choose between strategies emphasizing iron loading or GPX4 blockade based on a patient’s unique ncRNA profile [50,53,56]. These approaches align with broader efforts to target ncRNAs to overcome GBM chemoradiotherapy resistance and integrate naturally with ferroptosis-sensitizing strategies under development [25,26,72,73,81].

### 5.3. Combination Strategies with Current Therapies

Ferroptosis enables a departure from traditional cell-death pathways, offering non-redundant combinations with existing treatments. Combining ferroptosis inducers (erastin, RSL3, sulfasalazine, dihydroartemisinin) with TMZ has shown success in reversing acquired resistance in vitro in animal models [53,57,82]. Additionally, ferroptosis synergizes with radiotherapy and BH3 mimetics by bypassing the DNA repair mechanism and pushing glioma stem-like cells beyond their oxidative stress threshold [25,50,56]. From an immunological perspective, the release of damage-associated molecular patterns (DAMPs) during ferroptosis can stimulate dendritic cell activation and reprogram tumor-associated macrophages, providing a mechanistic rationale for combining induction therapy with immunotherapy [53,56,83]. FRLS that capture both the ferroptotic tone and immune landscape could help identify patients most likely to benefit from such multimodal regimens and guide trial design [53,70,71,84].

### 5.4. Delivery Challenges and Emerging Solutions

Translating these findings into clinical practice is hindered by significant delivery barriers, most notably the blood–brain barrier (BBB) and intratumoral heterogeneity [26,56,83]. Systemic administration of RNA-based therapies achieve intracranial penetration while minimizing peripheral toxicity [26,81]. Nanoparticle-mediated platforms—including lipid nanoparticles (LNPs), iron-containing cores, and engineered exosomes—have shown promise in orthotopic models for co-packaging ferroptosis inducers with ncRNA agents [26,49,53,83,85,86,87,88].

However, context-dependent roles of these molecules necessitate caution; spatial targeting and rigorous preclinical modeling are required to avoid neurotoxicity in healthy brain tissue [25,53,56]. Accordingly, ferroptosis and ncRNA-based strategies in GBM should at present be regarded as exploratory, preclinical concepts rather than ready-to-use therapeutic options, given unresolved blood–brain barrier, delivery, and neurotoxicity constraints.

### 5.5. Toward Precision Ferroptosis-Based GBM Therapy

In summary, ferroptosis-related ncRNAs provide a dual lever for the precision medicine framework in GBM. By utilizing FRLS scores as biomarkers for stratification and ncRNA-targeted agents to overcome therapeutic resistance, clinicians can potentially tune ferroptotic sensitivity [26,53,70,71] which could support escalation to clinical trials that combine TMZ/radiotherapy with ferroptosis inducers or ncRNA-targeted agents, rather than replacing standard therapy outright [57,71,89]. Future clinical strategies will likely integrate these signatures with advanced BBB-crossing delivery platforms moving beyond exploratory concepts toward the first wave of ferroptosis-based clinical trials in defined GBM subgroups [25,50,56,83,84].

## 6. Challenges and Future Directions

While ncRNA-mediated ferroptosis represents a fertile ground for glioblastoma research, the field remains in translational infancy. Addressing the biological and practical hurdles outlined below is essential before these insights can be deployed in a clinical setting [38,50,53,90,91].

### 6.1. Biological and Methodological Challenges

To date, our mechanistic understanding is restricted to a narrow subset of ncRNAs, with evidence largely derived from in vitro systems and limited xenograft models. Furthermore, the extreme genetic and metabolic heterogeneity of GBM—both within a single tumor and between primary and recurrent states—suggests that ncRNA–ferroptosis circuits may be highly context-dependent. Bulk transcriptomic approaches often obscure these nuances, masking cell type-specific roles in glioma stem cells versus the differentiated tumor mass or the immune compartment. A significant biological paradox lies in the bidirectional effect of ferroptosis on the immune microenvironment. While inducing ferroptosis can trigger immunogenic cell death and potentiate immunotherapy, nonselective inhibition of antioxidant systems (like GPX4) may inadvertently impair T-cell viability or damage healthy neural tissue [50,53,56,69,70,71,75,90,91,92].

Future efforts must utilize CRISPR/CRISPRi screens, spatial transcriptomics, and high-content ferroptosis assays to distinguish causal regulators from “passenger” correlations. Furthermore, the intersection of ncRNAs with the epigenetic and epitranscriptomic layers—including m^6^A-dependent RNA modifications—represents a burgeoning area of inquiry that may reveal how the ferroptosis threshold is set at a chromatin level. Robust in vivo models that recapitulate human GBM heterogeneity and the immune context, including patient-derived organoids and immune-competent mouse models, will be essential to test whether targeting specific ncRNA axes can safely tip the ferroptosis balance in tumors without unacceptable off-tumor toxicity [50,53,90,91,92,93,94,95,96].

### 6.2. Translational and Clinical Barriers

The translational pipeline for RNA-based therapeutics in GBM continues to face formidable obstacles regarding stability, specificity, and blood–brain barrier (BBB) penetration. While nanoparticle- and exosome-based platforms for co-delivering ferroptosis inducers and ncRNA mimics show promise, they require exhaustive pharmacokinetic and safety profiling. Moreover, current ferroptosis-related lncRNA signatures are largely retrospective and cohort-specific. For these tools to become clinically actionable, they must undergo prospective validation and harmonization across diverse patient populations [26,49,53,56,71,74,81,83,90,91].

### 6.3. A Path Toward Integrated Precision Therapy

Looking ahead, the integration of ferroptosis–ncRNA signatures with established molecular markers (e.g., IDH status, MGMT methylation) and immunogenomic features will enable precision stratification for multimodal therapy. The field must move from single-axis perturbations toward rational, multi-node strategies—simultaneously targeting ncRNA regulators and metabolic “brakes” like the GCH1–BH4 system. Guided by AI-assisted modeling to predict safe therapeutic windows, ncRNA-mediated ferroptosis has the potential to evolve from a mechanistic curiosity into a central pillar of personalized GBM treatment [50,56,71,74,91,92].

## 7. Conclusions

Noncoding RNA-mediated ferroptosis has emerged as a mechanistically distinct and clinically promising axis in glioblastoma. It resides at the vital intersection, bridging metabolic governance—including iron flux and lipid peroxidation—with the ncRNA-driven regulation of proliferation, invasion, stemness, and therapy resistance. Current experimental research has established that specific miRNAs, lncRNAs, and circRNAs—exemplified by miR 147a, TMEM161B AS1, circCDK14, and circLRFN5—can decisively modulate the ferroptosis threshold in GBM. By tuning iron export, ferritinophagy, the system Xc^−^/GPX4 axis, and BH4-dependent antioxidant pathways, these ncRNAs directly influence gliomal stem cell fitness and the response to temozolomide. In parallel, integrated muti-omics analyses of the TCGA and CGGA cohorts have identified robust ferroptosis-related lncRNA modules. These signatures do more than predict survival; they encode “ferroptotic tone” and reflect specific immune microenvironment states. By outperforming conventional clinicopathologic factors in prognostic stratification, they provide a systems-level framework that links complex ncRNA networks to patient outcome and treatment responsiveness [38,56,97].

Despite this progress, the field must now pivot to overcome several hurdles. These include the reliance on correlation-based data, the limited number of functionally validated targets and the persistent challenge of blood–brain barrier delivery. Moving forward, the integration of CRISPR-based functional screens, single-cell spatial transcriptomics, and advanced nanoparticle platforms will be essential. Such tools will allow the field to distinguish driver ncRNAs from “passengers”, refine ferroptosis ncRNA signatures into prospective biomarkers, and design combination regimens that exploit ferroptosis without harming normal neural tissue or anti-tumor immunity. Ultimately, integrating ncRNA-defined ferroptosis programs with established GBM markers (IDH, MGMT) will enable a truly personalized, ferroptosis-oriented treatment paradigm—addressing the core challenge of therapy resistance and offering a new horizon for patients with glioblastoma [39,98,99].

## Figures and Tables

**Table 1:** Representative ferroptosis-modulating noncoding RNAs in glioblastoma. The table summarizes expression patterns, principal targets/axes, effects on ferroptosis, and experimentally supported impacts on tumor biology and treatment response for selected miRNAs, lncRNAs, and circRNAs in GBM.

ncRNA	Primary Target(s)/Axis	Effect on Ferroptosis	Impact on Tumor Biology/Therapy	Reference
miRNA (miR 147a)	SLC40A1 (ferroportin)	Promotes iron overload and lipid peroxidation, induces ferroptotic death	Suppresses GBM cell growth and sensitizes cells to temozolomide in GBM cell lines and xenograft models	[35]
lncRNA (TMEM161B AS1)	miR 27a 3p targets FANCD2, and CD44 axis	Inhibits ferroptosis by sustaining DNA repair and antioxidant/stemness programs	Promotes proliferation, invasion, stemness, and TMZ resistance in GBM models; higher expression associated with poor outcome in glioma tissue	[37]
circRNA (circCDK14)	miR 3938 regulates PDGFRA and downstream SLC7A11/GPX4 signaling	Resists ferroptosis by maintaining PDGFRA signaling and GPX4/system Xc^−^ activity	Enhances proliferation, migration, invasion, and tumor growth in vitro and in vivo; high expression correlates with worse prognosis	[36]
circRNA (circLRFN5)	PRRX2 modulates GCH1–BH4 antioxidant pathway	Promotes ferroptosis via PRRX2 degradation and suppression of BH4 synthesis	Inhibits GSC self-renewal, invasion, and tumorigenicity in GBM models; ferroptosis-related effects are reversed by Ferrostatin-1	[34]

## Data Availability

The data presented in this study are available on request from the corresponding author.

## Abbreviations

The following abbreviations are used in this manuscript:

GBM	Glioblastoma
TMZ	Temozolomide
ncRNAs	noncoding RNAs
lncRNAs	long noncoding RNAs
